# Communication competence and resilience are modifiable factors for burnout of operating room nurses in South Korea

**DOI:** 10.1186/s12912-022-00985-0

**Published:** 2022-07-27

**Authors:** Eun Yeong Lee, Kyoung-ja Kim, Sangjin Ko, Eun Kyeung Song

**Affiliations:** 1 Department of Nursing, Ulsan University Hospital, Ulsan, South Korea; 2 Department of Nursing, College of Medicine, Inha University, Incheon, South Korea; 3 Department of Nursing, College of Medicine, University of Ulsan, Daehak-ro 93, Nam-gu, Ulsan, South Korea

**Keywords:** Occupational Stress, Resilience, Psychological, Communication, Burnout, Nurses

## Abstract

**Background:**

Burnout negatively impacts the personal and professional life of nurses. Job stress and resilience have been determined to be associated with nurse burnout. Given the importance of communication competence in operating room (OR) nurses, the associations of job stress, resilience, and communication competence with burnout have not been examined.

**Purpose:**

To determine the relationships of job stress, resilience, and communication competence to burnout of OR nurses in South Korea.

**Methods:**

This was a cross-sectional, descriptive study of 146 OR nurses. A series of self-reported questionnaires was used to assess job stress, resilience, communication competence, and burnout. Pearson correlation coefficient and a hierarchical linear regression were used for data analysis.

**Results:**

Communication competence was correlated with resilience (*r* = 0.65, *p* < .001) and burnout (*r* = -0.44, *p* < .001), and resilience was correlated with burnout (*r* = -0.48, *p* < .001). Resilience (β = -0.22, *p* = .027) and communication competence (β = -0.33, *p* < .001) were associated with burnout of OR nurses in a hierarchical linear regression (*F* = 6.28, *p* < .001).

**Conclusions:**

Increased resilience and communication competence were associated with lower burnout of perioperative nurses. To prevent and reduce burnout of OR nurses, it is necessary to develop and implement a program targeting for communication competence and resilience.

**Implications for nursing management:**

Nurse leaders should provide programs fostering communication competence and resilience to OR nurses and encourage them to actively participate in such job trainings.

## Introduction

Most nurses experienced burnout, which is a syndrome comprising emotional exhaustion, depersonalization, and a low level of perceived personal accomplishment [1]. Based on a recent meta-analysis [2] on the extent of compassion satisfaction and fatigue and burnout, the rate of nurse burnout was reported to be 52%. Regionally, it was reported that the nurse burnout rate was highest in Southeast Asia including South Korea [3], and Korean nurses revealed higher levels of burnout through a nationwide survey [4], which was a predictor of actual turnover [5]. Burnout has a variety of negative impacts on both the personal and professional life of nurses, and furthermore, nurse burnout has been directly or indirectly associated with patient safety, quality of care, and patient satisfaction [6–8].

Nurse burnout results from constant and chronic job stress [9], which is a dominant feature of nursing work, itself. Job stress including workload and demands has been one of the factors impacting on the emotional exhaustion of nurse burnout through a systematic review [7]. In particular, nurses working in the operating room (OR) experienced job stress emerging from tasks, collaboration or interaction, technology, and patient care related to time pressure [10, 11]. Due to the advanced, cutting-edge surgical instruments and equipment, more complex and specialized skills tend to be demanded for OR nurses, and thus, operating nurses can be more likely to experience a higher level of job stress [12]. Also, it was reported that job stress was a significant contributing factor to burnout in OR nurses [13].

As an alternative to relieve nurse burnout, there has been a growing attention to resilience, characterized by the ability to withstand adversity and bounce back from difficult life events [14]. Resilience was found to be negatively associated with nurse burnout [15, 16], indicating that resilience plays a role in protecting clinical nurses from burnout. Resilience was also a significant predictor of burnout, and was closely related to the job stress of operating nurses [13].

Communication competence is essential for OR nurses, who collaborate with other medical staff and perform nursing tasks within the operating team [17, 18]. However, the communication competence of OR nurses was not sufficient, compared to that of nurses working in general wards [19]. It was determined that job stress and resilience were associated with the communication competence of OR nurses [20], and communication competence was negatively correlated with burnout in OR nurses [21].

As seen above, job stress and resilience were associated with communication competence and burnout in OR nurses, and communication competence was related to burnout in OR nurses. However, the relationships of job stress, resilience, and communication competence to burnout of OR nurses have not been yet examined. This research gap may bring into a question of whether communication competence play a role in the relationships among job stress, resilience, and burnout of OR nurses. Given the importance of communication competence in OR nurses, this study aimed to determine whether job stress, resilience, and communication competence would be associated with burnout of nurses working at the OR.

## Methods

### Study design and setting

This was a cross-sectional, descriptive study to determine the relationships of job stress, resilience, and communication competence to burnout of OR nurses. Study participants were recruited from one tertiary medical center and three general hospitals at U metropolitan city, and one general hospital at C city, located in the southeastern area of Korea.

### Study subjects

Nurses working at the OR were eligible to participate in this study if they were a scrub nurse or a circulating nurse with longer than three months of nursing tasks in perioperative. New nurses with less than three months of clinical experience or during job training or nurse administrators/ managers at the OR were excluded from this study. Using the G*Power analysis software with a medium effect size of 0.15 and an expected R^2^ for job stress, resilience, and communication competence in the multiple linear regressions [22], α of 0.05, 95% power (1-β), and a total of up to 13 independent variables in the multiple linear regressions, the minimum sample size was estimated to be 119. Considering a 20% of dropout rate, a total of 150 subjects were recruited and 4 participants did not complete the questionnaire. Thus, data from a total of 146 participants were included in the final analysis.

### Measures

#### Job stress

The job stress questionnaire was originally developed to measure the degree of work stress of clinical nurses in Korea [23]. The modified version of the job stress questionnaire for the OR nurses [24] was used to assess job stress. This instrument consists of 10 stressor domains including work overload, interpersonal relationship and job conflict with surgeons, lack of expertise and skills, emotional burden on medical limit, duty schedule, professional role conflict, interpersonal relationship related to task, work condition of nurses, and physical environment within the OR. It comprises a total of 47 items asking how much stress one perceives in the following cases, and each item is rated on a scale of 1 (not at all) to 5 (very heavily). The total score is the sum of the ratings for the 47 items with a possible range of 47 to 235, with a higher score reflecting more severe job stress. Its reliability was previously established with a Cronbach’s α coefficient of 0.94 [24], and in this study, the Cronbach’s α coefficient was 0.97.

#### Resilience

The Connor-Davison Resilience Scale [25] was used to measure the resilience of OR nurses. Each item is rated on a 5-point scale (0 to 4), with a higher score indicating a greater resilience. The Korean version of the Connor-Davison Resilience Scale was previously verified to assess resilience with high internal consistency, test–retest reliability, and convergent validity in the general population [26]. The Cronbach’s α coefficient for the Korean version of the Connor-Davison Resilience Scale was 0.93, which had a five-factor structure including hardiness, persistence, optimism, support, and spiritual in nature [26]. In this study, the Cronbach’s α coefficient was 0.93.

#### Communication competence

Communication competence was defined as knowledge, skills, and motivation for communication involving appropriates and effectiveness [27]. To assess the communication competence of nurses working at the OR, the revised Korean version of the Global Interpersonal Communication Competence (GICC) scale [27] was used. The revised scale consists of 15 items regarding self-disclosure, empathy, social relaxation, assertiveness, concentration, interaction management, expressiveness, supportiveness, immediacy, efficiency, social appropriateness, conversational coherence, goal detection, responsiveness, and noise control [28]. It is self-rated on a 5-point scale (1 = ‘not true at all’ to 5 = ‘true nearly all the time’) according to the extent to which they agree with each item. The total score is achieved by summing all responses, and ranges from 15 to 75, with a higher score indicating greater communication competence. The Cronbach’s α coefficient for the revised Korean version of the GICC scale was 0.83 [28], and in this study, the Cronbach’s α coefficient was 0.88.

#### Burnout

Maslach and Jackson (1981) developed the Maslach Burnout Inventory (MBI) to assess burnout [29], which refers to a syndrome of emotional exhaustion and cynicism that occurs frequently among individuals with interpersonal work. To assess burnout of OR nurses in this study, the modified Korean version of the MBI [30] was used, which contains 3 subscales: 9 items of emotional exhaustion, 5 items of depersonalization, and 8 items of personal accomplishment. Each item was scored from 1 (not true at all) to 5 (true nearly all the time), with a higher score indicating more severe burnout. The Cronbach’s α coefficient for the modified Korean version of the MBI was 0.95 [30], and in this study, the Cronbach’s α coefficient was 0.90.

#### General characteristics

General characteristics were obtained with a self-reported questionnaire from participants, including age, gender, marital status, religion, education level, clinical career at OR, working pattern, monthly income, whether a nurse wished to be assigned to an OR, and number of turnovers.

### Data collection

Prior to data collection, the primary investigator explained the purpose and procedures for this study. Written informed consent was obtained from each participant. A series of questionnaires was provided to subjects who were willing to participate in this study. It lasted approximately 15–20 min. Each completed questionnaire was sealed in an envelope along with the written consent form. Data were collected from August 10 to August 31, 2020.

### Data analysis

SPSS for Windows 24.0 (IBM, Armonk, New York, USA) was used for all data analyses; *p* < 0.05 was considered significant throughout. Means with standard deviations, and numbers with frequencies are shown for descriptive statistics. Independent t-tests and analysis of variance (ANOVA) were used to determine differences in job stress, resilience, communication competence, and burnout according to general characteristics. The Pearson correlation coefficient was used to determine the correlation between job stress, resilience, communication competence, and burnout. The assumption of no multicollinearity was kept in this study with a tolerance value of 0.515 to 0.986, and a variance inflation factor of 1.015 to 1.944 (close to 1–2). A hierarchical linear regression analysis was conducted to determine the relationships of job stress, resilience, and communication competence to burnout, after controlling for variables that were significantly related to job stress, resilience, communication competence, and burnout in bivariate analysis.

### Ethical considerations

The research proposal was approved by the Institutional Review Board of a tertiary medical center in U metropolitan city to ensure that the rights of human subjects were protected (U** 2020–07-003–002). Written informed consent was obtained from all participants.

## Results

### General characteristics of participants

The general characteristics of a total of 146 perioperative nurses are shown in Table 1. Eighty-six percent of OR nurses were women, and more than half were younger than 30 years. Nurses with longer than five years of clinical career at OR accounted for 47.3%, those with three shifts accounted for 56.8%, and those with monthly income for less than 3 million Korean won accounted for more than 70%.

**Table 1:** General Characteristics of Study Subjects (*N* = 146)

Variables	Categories	n (%)
Gender	Male	21 (14.0)
	Female	125 (86.0)
Age (years)	< 30	83 (56.8)
	≥ 30	63 (43.2)
Marital status	Single	102 (69.9)
	Married	44 (30.1)
Religion	Yes	49 (33.6)
	None	97 (66.4)
Education level	Below a bachelor’s degree	46 (31.5)
	With a bachelor’s degree	89 (61.0)
	In Master’s program or higher	11 (07.5)
Clinical career at operating room (years)	1 ~ < 3	41 (28.1)
	3 ~ < 5	36 (24.7)
	5 ~ < 10	35 (24.0)
	≥ 10	34 (23.3)
Working pattern	Three shift	83 (56.8)
	Two shift	11 (07.5)
	None shift	52 (35.6)
Monthly income (1,000 won)	< 2,500	36 (24.7)
	2,500 ~ 3,000	70 (47.9)
	3,000 ~ 3,500	19 (13.0)
	3,500 ~ 4,000	14 (09.6)
	> 4,000	7 (04.8)
Department assignment	Matched to a nurse’s wish	72 (49.3)
	As assigned	74 (50.7)
Number of turnover	None	93 (63.7)
	Once	40 (27.4)
	≥ Twice	13 (08.9)

### Differences in job stress, resilience, communication competence, and burnout according to the general characteristics of participants

Women had higher levels of job stress (t = -2.81, *p* = 0.006) but lower levels of communication competence (t = 2.47, *p* = 0.015) than men. Nurses under 30 years old had a lower level of resilience than nurses over 30 years old (t = -2.03, *p* = 0.044). Nurses with spouse had a higher level of resilience (t = -2.29, *p* = 0.023) and a lower level of emotional exhaustion of burnout (t = 2.02, *p* = 0.045) than those who were single. Regarding education level, nurses with a bachelor’s degree or higher had higher levels of job stress (t = 2.16, *p* = 0.033), lower level of total burnout scores (t = 2.14, *p* = 0.034), and lower levels of emotional exhaustion (t = 2.54, *p* = 0.012) compared to nurses with education below a bachelor’s degree. There was a significant difference in emotional exhaustion of burnout by working pattern (t = 2.21, *p* = 0.028) and in resilience (t = -2.14, *p* = 0.034) by monthly income. Nurses who wanted to work in the OR had higher levels of resilience (t = 2.45, *p* = 0.016) and communication competence (t = 2.94, *p* = 0.004), compared to nurses who were assigned to an OR without considering their wishes. There was no significant difference in job stress, resilience, communication competence, or burnout according to religion, clinical career at OR, or number of turnovers (Tables 2 and 3).

**Table 2:** Differences in Job Stress, Resilience, Communication Competence by General Characteristics (*N* = 146)

Variables	Categories	Job stress		Resilience		Communication competence	
		Mean ± SD	t or F (*p* value)	Mean ± SD	t or F (*p* value)	Mean ± SD	t or F (*p* value)
Gender	Male	146.90 ± 24.56	-2.81 (.006)	55.10 ± 7.48	1.69 (.093)	55.76 ± 6.44	2.47 (.015)
	Female	166.78 ± 30.72		50.78 ± 11.28		51.67 ± 7.10	
Age (years)	< 30	160.12 ± 31.79	-1.73 (.086)	49.82 ± 10.07	-2.03 (.044)	51.69 ± 7.28	-1.12 (.266)
	≥ 30	168.92 ± 28.55		53.48 ± 11.66		53.02 ± 6.91	
Marital status	Single	161.41 ± 31.44	-1.51 (.133)	50.06 ± 10.79	-2.29 (.023)	52.30 ± 7.68	0.12 (.911)
	Married	169.73 ± 28.19		54.50 ± 10.64		52.16 ± 5.75	
Religion	None	164.51 ± 29.78	0.33 (.746)	50.98 ± 10.18	-0.65 (.516)	52.02 ± 7.30	-0.57 (.570)
	Yes	162.76 ± 32.58		52.22 ± 12.27		52.73 ± 6.83	
Education level	Below a bachelor’s degree	155.96 ± 34.80	2.16 (.033)	50.02 ± 12.28	1.04 (.303)	52.20 ± 6.32	-0.13 (.894)
	With a bachelor’s degree or higher	167.58 ± 27.97		52.03 ± 10.21		52.39 ± 8.71	
Clinical career at the operating room (years)	1 ~ < 3	154.32 ± 32.81	2.09 (.104)	49.15 ± 10.05	1.38 (.252)	51.51 ± 7.56	0.82 (.484)
	3 ~ < 5	165.89 ± 32.47		50.50 ± 9.03		52.47 ± 6.85	
	5 ~ < 10	166.40 ± 30.92		52.60 ± 11.36		53.74 ± 8.00	
	≥ 10	170.85 ± 23.32		53.82 ± 12.88		51.41 ± 5.89	
Working pattern	None shift	159.00 ± 29.99	1.45 (.150)	50.44 ± 9.62	0.79 (.433)	51.63 ± 7.51	0.79 (.432)
	Two or three shift	166.64 ± 30.82		51.93 ± 11.56		52.61 ± 6.93	
Monthly income (1,000 won)	Below 3,000	164.84 ± 30.16	0.59 (.556)	50.23 ± 7.03	-2.14 (.034)	51.88 ± 7.17	-1.06 (.293)
	≥ 3,000	161.48 ± 32.16		54.50 ± 12.56		53.28 ± 7.03	
Department assignment	Matched to a nurse’s wish	159.38 ± 30.70	-1.78 (.077)	53.60 ± 11.92	2.45 (.016)	53.97 ± 7.80	2.94 (.004)
	As assigned	168.34 ± 30.14		49.26 ± 9.37		50.59 ± 6.01	
Number of turnover	None	165.70 ± 31.61	0.93 (.354)	51.01 ± 10.88	-0.57 (.572)	51.84 ± 6.62	-0.95 (.346)
	More than once	160.72 ± 28.89		52.08 ± 10.99		53.00 ± 7.97

**Table 3:** Difference in Burnout by General Characteristics (*N* = 146)

Variables	Categories	Total		Emotional exhaustion		Depersonalization		Personal accomplishment	
		Mean ± SD	t or F (*p* value)	Mean ± SD	t or F (*p* value)	Mean ± SD	t or F (*p* value)	Mean ± SD	t or F (*p* value)
Gender	Male	52.14 ± 14.40	-1.72 (.088)	25.67 ± 7.00	-0.99 (.334)	9.00 ± 3.63	-1.89 (.061)	17.48 ± 6.55	-1.20 (.230)
	Female	56.65 ± 10.51		27.23 ± 4.80		10.53 ± 3.39		18.89 ± 4.67	
Age (years)	< 30	56.87 ± 10.46	1.07 (.284)	27.42 ± 4.98	1.11 (.267)	10.51 ± 3.48	0.79 (.429)	18.94 ± 4.87	0.71 (.480)
	≥ 30	54.86 ± 12.10		26.46 ± 5.40		10.05 ± 3.44		18.35 ± 5.13	
Marital status	None	57.15 ± 11.52	1.90 (.059)	27.57 ± 5.37	2.02 (.045)	10.55 ± 3.49	1.28 (.201)	19.03 ± 5.18	1.28 (.204)
	Yes	53.34 ± 10.04		25.70 ± 4.47		9.75 ± 3.34		17.89 ± 4.43	
Religion	None	55.82 ± 11.04	-0.27 (.791)	26.89 ± 5.23	-0.39 (.694)	10.09 ± 3.41	-1.06 (.291)	18.85 ± 4.97	0.55 (.586)
	Yes	56.35 ± 11.63		27.24 ± 5.09		10.73 ± 3.55		18.37 ± 5.04	
Education level	Below a bachelor’s degree	53.11 ± 11.31	2.14 (.034)	25.43 ± 5.24	2.54 (.012)	9.70 ± 3.52	1.46 (.147)	17.98 ± 4.82	1.17 (.246)
	With a bachelor’s degree or higher	57.33 ± 10.95		27.73 ± 5.00		10.59 ± 3.41		19.01 ± 5.04	
Clinical career at the operating room (years)	1 ~ < 3	56.83 ± 10.01	0.46 (.711)	27.17 ± 4.77	0.90 (.446)	10.32 ± 3.16	0.61 (.607)	19.34 ± 4.86	0.36 (.782)
	3 ~ < 5	57.14 ± 12.18		28.06 ± 5.77		10.86 ± 4.03		18.22 ± 4.99	
	5 ~ < 10	54.40 ± 11.84		26.17 ± 5.35		9.74 ± 3.41		18.49 ± 5.28	
	≥ 10	55.44 ± 11.09		26.56 ± 4.81		10.29 ± 3.25		18.59 ± 4.93	
Working pattern	None shift	56.13 ± 11.21	-0.11 (.914)	25.75 ± 5.29	2.21 (.028)	10.96 ± 3.62	-1.71 (.090)	19.42 ± 5.16	-1.34 (.184)
	Two or three shift	55.93 ± 11.26		27.70 ± 5.00		9.95 ± 3.33		18.28 ± 4.86	
Monthly income (1,000 won)	Below 3,000	56.75 ± 11.17	1.03 (.192)	27.51 ± 5.05	1.93 (.056)	10.39 ± 3.54	0.45 (.656)	18.85 ± 4.93	0.65 (.519)
	≥ 3,000	54.03 ± 11.20		25.68 ± 5.33		10.10 ± 3.27		18.25 ± 5.15	
Department assignment	Matched to a nurse’s wish	54.58 ± 11.17	-1.51 (.132)	26.26 ± 5.21	-1.72 (.087)	10.08 ± 3.33	-0.77 (.440)	18.24 ± 5.10	-1.08 (.284)
	As assigned	57.38 ± 11.14		27.73 ± 5.07		10.53 ± 3.58		19.12 ± 4.85	
Number of turnover	None	55.86 ± 11.47	-0.20 (.843)	27.05 ± 5.46	0.15 (.885)	10.39 ± 3.52	0.36 (.716)	18.42 ± 4.99	-0.85 (.395)
	More than once	56.25 ± 10.83		26.92 ± 4.68		10.17 ± 3.37		19.15 ± 4.96	

### Correlation between job stress, resilience, communication competence, and burnout

The average scores of job stress, resilience, communication competence, and burnout of nurses in this study were 3.49 ± 0.65, 2.34 ± 0.51, 3.48 ± 0.48, and 2.55 ± 0.51, respectively. Job stress was not correlated with burnout (*r* = 0.13, *p* = 0.098), whereas job stress was significantly correlated with two subscales of burnout: emotional exhaustion (*r* = 0.16, *p* = 0.049) and depersonalization (*r* = 0.20, *p* = 0.018). Communication competence had a positive correlation with resilience (*r* = 0.65, *p* < 0.001) but a negative correlation with burnout (*r* =—0.44, *p* < 0.001). There was a negative correlation between resilience and burnout (*r* =—0.48, *p* < 0.001) (Table 4).

**Table 4:** Correlation among Job Stress, Resilience, Communication Competence and Burnout (*N* = 146)

Variables	Job stress	Resilience	Communication competence	Burnout
	r(*p*)			
**Job stress**	1			
**Resilience**	- 0.04 (.599)	1		
**Communication competence**	- 0.09 (.308)	0.65 (< .001)	1	
**Burnout**	0.13 (.098)	- 0.48 (< .001)	- 0.44 (< .001)	1

### Factors associated with burnout of or nurses

Age, gender, marital status, education level, working pattern, monthly income, and department assignment among general characteristics were entered in step 1, and then, job stress, resilience, and communication competence were entered in step 2 of a hierarchical multiple linear regression. As a result of the hierarchical multiple linear regression analysis, education level (β =—0.17, *p* = 0.031), resilience (β =—0.22, *p* = 0.027), and communication competence (β =—0.33, *p* < 0.001) were associated with burnout of OR nurses. These three factors explained 27% of the variance in burnout in OR nurses (*F* = 6.28, *p* < 0.001) (Table 5).

**Table 5:** The Link of Job Stress, Resilience and Communication Competence to Burnout in Hierarchical Linear Regression (*N* = 146)

Variables	Burnout											
	Total			Emotional exhaustion			Depersonalization			Personal accomplishment		
	β	t	*p*	β	t	*p*	β	t	*p*	β	t	*p*
Step 1												
Female, gender (*vs.* male)	0.05	0.66	.511	0.04	0.45	.653	0.08	0.99	.322	0.02	0.25	.806
Age, ≥ 30 years (*vs.* < 30)	0.07	0.80	.425	0.06	0.59	.556	0.02	0.22	.829	0.09	1.01	.313
Married (*vs*. single)	- 0.13	- 1.45	.150	- 0.13	- 1.40	.163	- 0.10	- 1.07	.289	- 0.08	- 0.92	.359
Below a bachelor’s degree (*vs*. with a bachelor’s degree or higher)	- 0.17	- 2.19	.031	- 0.17	- 2.11	.036	- 0.10	- 1.19	.238	- 0.13	- 1.72	.087
None shift (*vs*. two or three shift)	0.00	0.02	.982	- 0.17	- 2.05	.043	0.15	1.90	.060	0.07	0.95	.343
Monthly income, < 3,000 (1,000 won) (*vs*. ≥ 3,000 (1,000 won))	- 0.03	- 0.35	.731	- 0.05	- 0.53	.594	- 0.02	- 0.22	.829	- 0.00	- 0.02	.986
Department assignment matched to a nurse’s wish (*vs.* as assigned)	- 0.02	- 0.20	.843	0.04	0.52	.605	- 0.04	- 0.54	.589	- 0.05	- 0.64	.522
Step 2												
Job stress	0.07	0.85	.398	0.08	1.01	.314	0.17	1.99	.048	- 0.06	- 0.74	.463
Resilience	- 0.22	- 2.23	.027	- 0.19	- 1.78	.078	- 0.09	- 0.88	.383	- 0.24	- 2.46	.015
Communication competence	- 0.33	- 3.36	.001	-0.14	-1.34	.183	- 0.27	- 2.52	.013	- 0.41	- 4.32	< .001
Overall	Adjusted *R*^2^ = 0.27, change in adjusted *R*^2^ = 0.22, *F* = 6.28, *p* < .001			Adjusted *R*^2^ = 0.15, change in adjusted *R*^2^ = 0.07, *F* = 3.57, *p* < .001			Adjusted *R*^2^ = 0.15, change in adjusted *R*^2^ = 0.11, *F* = 3.56, *p* < .001			Adjusted *R*^2^ = 0.31, change in adjusted *R*^2^ = 0.30, *F* = 7.61, *p* < .001		

As a result of confirming factors associated with the subscales of burnout through a hierarchical multiple linear regression, education level (β =—0.17, *p* = 0.036) and working pattern (β =—0.17, *p* = 0.043) were associated with emotional exhaustion. Job stress (β = 0.17, *p* = 0.048) and communication competence (β =—0.27, *p* = 0.013) were associated with depersonalization. Communication competence (β =—0.41, *p* < 0.001) and resilience (β =—0.24, *p* = 0.015) were associated with personal accomplishment.

## Discussions

The most compelling finding of this study was that communication competence was adversely associated with burnout of OR nurses after controlling for other factors related to burnout. This indicates that communication competence can play an important role in protecting OR nurses from burnout. Every one-point increase in communication competence score accounted for an 11% decrease in total burnout score in our study. This finding aligns with a previous study, demonstrating that communication competence was highly correlated with burnout among OR nurses [21]. In particular, better communication within the OR team or being acknowledged for their performance in team dynamics was identified as a significant factor for perioperative nurses [17, 31]. Educational programs for promoting the communication competence of nurses and medical staff have been reported to show positive effects on communication and collaboration [32, 33]. Therefore, it is necessary to develop and implement an effective program to improve the communication competence of OR nurses. In addition, its effectiveness should be verified to decrease the burnout of perioperative nurses.

The burnout level of OR nurses in our study was similar to them of some studies using the same tool to measure burnout of OR nurses [13, 34, 35]. When nurse burnout was moderate or higher, turnover intention increased by about 1.5 times in a national survey of Korea [4]. Also, burnout was found to have the greatest impact on turnover intention of OR nurses in South Korea [35]. Particularly, our study showed the highest level of emotional exhaustion, which was a predictor of turnover intention among 3 subscales of burnout [36]. However, only a few studies have focused on burnout of OR nurses [35]. Future studies are needed to determine factors influencing burnout of OR nurses and establish preventive specific strategies to decrease burnout.

In this study, increased resilience was significantly associated with decreased burnout in OR nurses. This was consistent with findings of prior investigations showing a link of resilience to burnout of perioperative nurses [13], clinical nurses [16], and nurses in intensive care units [37]. Particularly, resilience predicted personal accomplishment among subscales of burnout in our study. This reflects that a perioperative nurse who tends to recover well when faced with a variety of difficulties and professional challenges is likely to have greater competence and better performance at work. Also, resilience was positively correlated with communication competence in our study. This is similar to a finding of a previous study regarding the relation of resilience to communication competence of OR nurses [20]. Taken together, it would be better to investigate whether resilience plays a moderating role in the link between communication competence and burnout of perioperative nurses in a future study. Dr. Mearler and colleagues reported that a 12-week resilience training program for intensive care unit nurses resulted in improved resilience but not in decreased burnout through a randomized controlled trial [38]. More recently, Dr. Grabbe et al. showed that resilience improved over time in a group of clinical nurses with a 3-h community resiliency training; however, the burnout score did not decrease over time [39]. Accordingly, a well-designed intervention program would be developed to enhance resilience and decrease burnout of OR nurses. A program for OR nurses to promote resilience as well as communication competence would be implemented as part of the job training, and further, efforts should be made to establish this organizational culture in the clinical area.

Job stress was not associated with resilience, communication competence, or total burnout score, but job stress predicted depersonalization among subscales of burnout in our study. This indicates that perioperative nurses who experience more heavy job stress are likely to have a negative and detached response to other people. Similar findings could be found in a theoretical review of burnout in nursing [8], demonstrating that stress emerging from heavy workload predicted depersonalization or cynicism. Also, it was reported that nurses with a high workload are likely to have a three times higher risk for depersonalization than those with a low workload in a systematic review of the work environment and burnout symptoms [7]. Furthermore, it was identified that job stress predicted emotional exhaustion, which in turn predicted depersonalization [40–42]. Therefore, additional research is required to determine the relationship of job stress to subscales of burnout of perioperative nurses.

Regarding education level, perioperative nurses with a bachelor’s degree or higher had a greater level of burnout compared to those with an education level below a bachelor’s degree in our study. However, it was determined that a higher education level was related to a lower rate of burnout in a meta-analysis of the extent of nurse burnout [2]. In that meta-analysis [2], the percentage of a nurse with a bachelor’s degree or higher was 55.26%, which was lower than that (68.5%) in our study. In addition, the sample population of that meta-analysis [2] included nurses from pediatrics, medical or surgical units, palliative care units, and intensive care units, but not perioperative nurses. Therefore, further study is recommended to explore whether education level is associated with burnout in a larger sample size of perioperative nurses.

Our study has several limitations. A cross-sectional study design was used in our study and all variables were measured at one time-point, which might not reflect the causal relationship among resilience, communication competence, and burnout. Future work needs to determine the impacts of resilience and communication competence on burnout through randomized controlled trials. Our samples were predominantly females and had a bachelor’s degree or higher. In addition, data were collected from several general hospitals and a tertiary medical center in South Korea. Therefore, our findings warrant further study to confirm the relationships among job stress, resilience, communication competence, and burnout in a more diverse setting of nurses.

### Conclusions

Our study showed that greater resilience and communication competence were associated with decreased burnout of perioperative nurses. Higher job stress and lower communication competence predicted greater risk for depersonalization, and greater resilience and communication competence predicted lower risk for decreased personal accomplishment among subscales of burnout. A program targeting improving the resilience and communication competence of perioperative nurses would be designed and implemented to reduce burnout. Furthermore, it is recommended that such a program would be included in the job training for nurses working in the OR.

## Implications for nursing management

Good news is that both resilience and communication competence are modifiable factors for relieving burnout in perioperative nurses. Resilience training or communication promoting programs could be utilized to mitigate burnout in OR nurses, and more nurses would actively engage in such trainings. In addition, such education programs should be developed available in a variety of ways from online offerings to hands-on trainings. Of note is that organizational effort should be first established within the purview of nursing departments to provide such programs to nurses as a part of job training. Nurse leaders or nurse managers would play a pivotal role in implementing such trainings to improve the work environment and build up the organizational culture. As nurturing collegial relationships between nurses and surgeons within the team dynamic was of high importance at the OR, specific strategies learned from such trainings can help OR nurses protect from burnout.

## Data Availability

The authors confirm that all the relevant data are included in the article. All data supporting the findings of this study are available from the corresponding author on reasonable request.

## Abbreviations

ANOVA: Analysis of variance
GICC: Global Interpersonal Communication Competence
MBI: Maslach Burnout Inventory
OR: Operating room
